# Social network analysis and the implications for Pontocaspian biodiversity conservation in Romania and Ukraine: A comparative study

**DOI:** 10.1371/journal.pone.0221833

**Published:** 2020-10-23

**Authors:** Aleksandre Gogaladze, Niels Raes, Jacobus C. Biesmeijer, Camelia Ionescu, Ana-Bianca Pavel, Mikhail O. Son, Natalia Gozak, Vitaliy V. Anistratenko, Frank P. Wesselingh

**Affiliations:** 1 Naturalis Biodiversity Center, Leiden, The Netherlands; 2 Institute of Environmental Sciences, Leiden University, Leiden, The Netherlands; 3 NLBIF–Netherlands Biodiversity Information Facility, Leiden, The Netherlands; 4 WWF Romania, Bucharest, Romania; 5 Constanta Branch of the National Institute for Research and Development on Marine Geology and Geo-ecology–GeoEcoMar, Constanta, Romania; 6 Institute of Marine Biology, National Academy of Sciences of Ukraine, Odessa, Ukraine; 7 NGO "Ecoaction", Kyiv, Ukraine; 8 Department of Invertebrate Fauna and Systematics, Schmalhausen Institute of Zoology, National Academy of Sciences of Ukraine, Kiev, Ukraine; 9 Department of Earth Sciences, Utrecht University, Utrecht, The Netherlands; University of Sao Paulo, BRAZIL

## Abstract

Romania and Ukraine share the Black Sea coastline, the Danube Delta and associated habitats, which harbor the endemic, aquatic Pontocaspian biota. Currently, this biota is diminishing both in numbers of species and their abundance because of human activities, and its future persistence strongly depends on the adequacy of conservation measures. Romania and Ukraine have a common responsibility to address the conservation of Pontocaspian biodiversity. The two countries, however have different socio-political and legal conservation frameworks, which may result in differences in the social network structure of stakeholder institutions with different implications for Pontocaspian biodiversity conservation. Here, we study the social network structure of stakeholder organizations involved in conservation of Pontocaspian biodiversity in Romania and the implications of network structure for conservation outcomes. Then we compare the findings from Romania to an earlier similar study from Ukraine. We apply a mix of qualitative and quantitative social network analysis methods to combine the content and context of the interactions with relational measures. We show that Pontocaspian biodiversity plays a minor and mostly incidental role in the inter-organizational interactions in Romania. Furthermore, there is room for improvement in the network structure through e.g. more involvement of governmental and nongovernmental organizations and increased motivation of central stakeholders to initiate conservation actions. Social variables, such as lack of funding, hierarchical, non-inclusive system of conservation governance and continuous institutional reforms in the public sector are consequential for the network relations and structure. Social network of stakeholders in Ukraine is more connected and central stakeholders utilize their favorable positions. However, neither in Ukraine is the Pontocaspian biodiversity a driver of organizational interactions. Consequently, both networks translate into sub-optimal conservation actions and the roads to optimal conservation are different. We end with sketching out conservation implications and recommendations for improved national and cross-border conservation efforts.

## Introduction

Romania and Ukraine hold an important part of the Pontocaspian (PC) habitats in the Northern part of the Black Sea Basin, which harbor aquatic PC community [1–3]. The PC biota comprises endemic flora and fauna including mollusks, crustaceans, planktonic groups (e.g. dinoflagellates and diatoms) and fish species [3–5]. Currently, PC species numbers and abundances are in decline as a result of human activities and their future persistence strongly depends on the adequacy of conservation measures [3, 6, 7]. The distribution of PC species in Romania is limited to the Razim-Sinoe-Babadag lake complex [8, 9], the area along the Danube River and the Black Sea coastal zone, which together form the Danube Delta and have the status of Biosphere Reserve. In Ukraine, PC communities occur in the coastal lakes, deltas and estuaries from the Danube Delta in the south to the Dnieper estuary in the north and in the north-eastern part of the Sea of Azov [10–12]. The two countries share the responsibility of conserving the PC habitats and the associated threatened biota [9, 11, 13, 14]. However, they have different socio-political settings and histories. Romania is a member of the European Union (EU) since 2007, thus complying with the EU environmental policy, whereas Ukraine is an EU-associated country since 2017. Being part of the EU, Romania experiences continuous adjustments in the institutional alignment [15] and a transformation of governance systems from authoritative state, to democratic and inclusive, multi-stakeholder systems [16]. This may result in different social environment in Romania to deal with biodiversity conservation issues compared to Ukraine [17].

In both countries Pontocaspian species are threatened and conservation measures are urgently required. In the past 30 years, the number, abundance and distribution ranges of PC species have decreased dramatically in Romania as a result of human influence [8, 9]. In Ukraine, PC species are declining as a result of habitat fragmentation caused by river damming and deep sea shipping lane constructions [18, 19]. Some of the PC species (e.g. some mollusk and sturgeon species) are of national concerns in both countries—they are recognized to be threatened and in need of conservation [8, 11, 13, 14]. Yet, indications exist that strong conservation measures are not in place to preserve these species and populations continue to decrease in both countries [8–10].

Biodiversity conservation is a complex task which involves different interests of various actors. Therefore, it is crucial that all types of stakeholder organizations are participating and interact at different stages of the process [20]. Effective exchange of scientific information, knowledge and conservation management experiences between stakeholder organizations determine the positive outcomes for biodiversity conservation [21–23]. Social network analysis (SNA) is a commonly used tool to map and quantify these interactions. Social networks, defined as the sets of relationships among the stakeholder organizations, work as channels that facilitate the flow of information and provide opportunities for joint action and collaboration [24–26]. SNA uses a combination of mathematical formulae and models to describe and quantify the existing links among organizations [23]. In recent years, SNA has gained increased attention across a variety of domains including biodiversity conservation [27–29] and proved to be very informative for conservation planning [30].

The structure of a social network has implications for biodiversity conservation. Social networks can vary in their properties, for example, in the number of connections, the structural position of individual stakeholders or the frequency of interactions between stakeholders. There is no single network structure that will be most beneficial in all contexts [31, 32]. There are, however, certain network properties which are suggested to facilitate effective management of natural resources and effective conservation of biodiversity. For example, a high number of connections in a network was shown to enable improved transfer of information relevant to biodiversity conservation [33, 34]. Similarly, strong, i.e. frequent connections are desirable for effective conservation as they indicate high levels of trust [35–38]. Weak, or less frequent connections on the other hand, facilitate the transfer of novel information as they tend to connect dissimilar actors [39, 40]. Furthermore, networks in which only one or a limited number of organizations have a central position (holding the majority of relational ties) are more effective for quick mobilization of resources and decision making in the initial phase of conservation action [41, 42]. On the contrary, networks with more organizations in a central position are more suitable for long-term environmental planning and complex problem-solving [35]. In summary, whether a network is optimal or not depends on the local context, the organizations that are involved, and the phase of the conservation process [35, 43, 44].

Merely the structural analysis of a network may not be sufficient to fully understand all the processes and dynamics within the network. Therefore, a qualitative analysis of the data provided by the stakeholders is very important to inform and explain the results of the SNA [45]. Qualitative data on the nature and content of reported interactions, as well as the additional social variables, such as the funding schemes, stability and functioning of organizations, the implementation capacity and the governance arrangements, amongst others provide a deeper understanding of how the network functions and translates into conservation action [44]. Combining a quantitative structural analysis of the network data with a qualitative analysis of the interactions is referred to as the mixed-method approach [29, 46].

Here we employ the mixed-method approach to analyze the information sharing network of stakeholders, which are involved in Pontocaspian biodiversity conservation in Romania and compare this network to the similar stakeholder network of Ukraine, which was studied using the same analytical approach [17]. This study is part of the Horizon 2020 ‘Pontocaspian Biodiversity Rise and Demise’ (PRIDE) program (http://www.pontocaspian.eu/) which was designed to generate scientific knowledge on PC biota and guide effective conservation action. We assess whether the different socio-political contexts in Romania and Ukraine result in differences in the social network structure of stakeholders, the content of the interactions and the external social variables which may help or hinder the functioning of the network. Importantly, we aim to identify how differences and/or similarities in the two networks translate into PC biodiversity conservation. We conclude the paper with recommendations for improved national and cross-border conservation efforts.

## Materials and methods

### Stakeholder identification and prioritization

**Fig 1:**
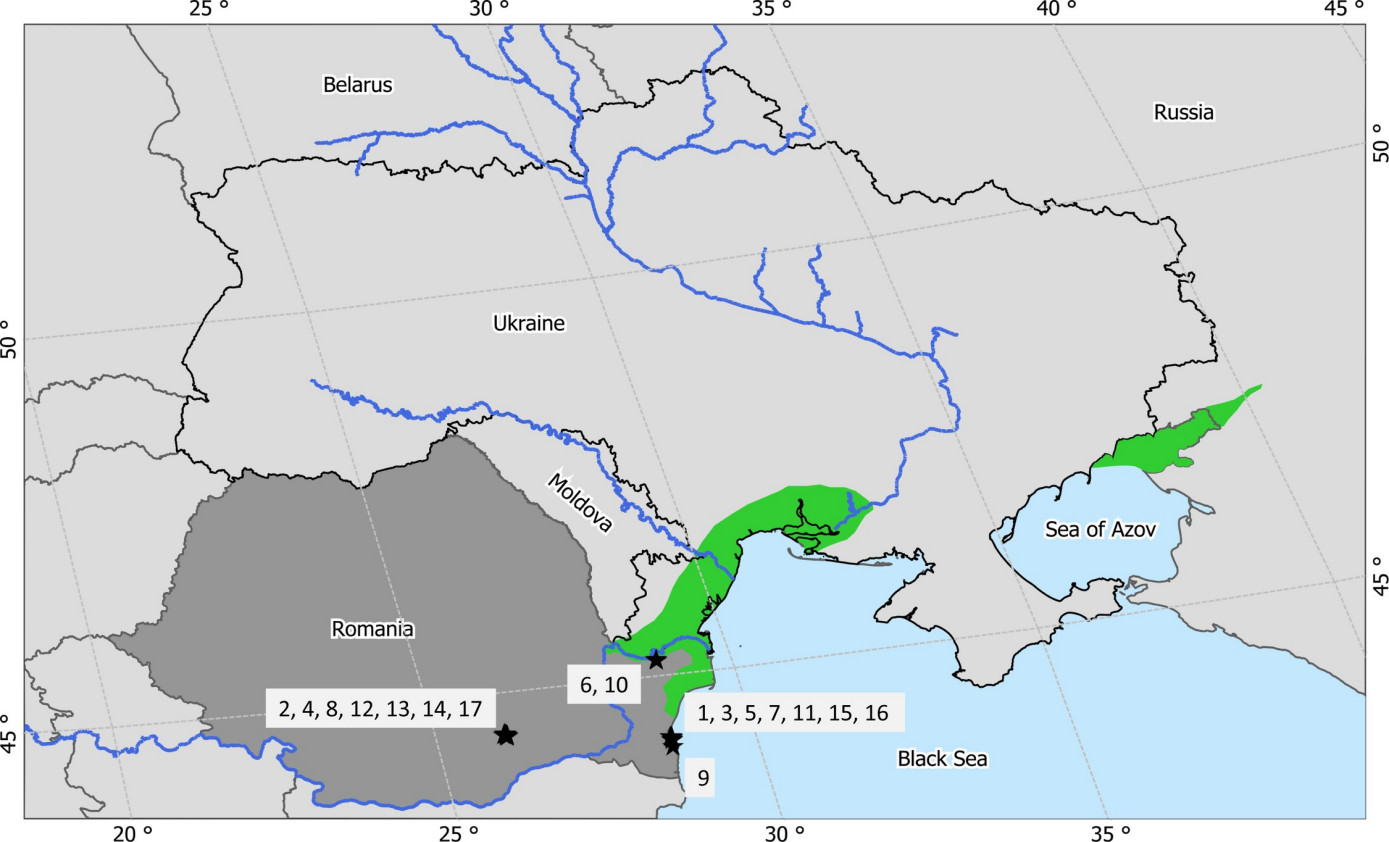
Map of the study area. Black stars on the map represent the stakeholder institutions (see IDs in Table 1). Green shading indicates major Pontocaspian habitats.

We applied the whole network analysis approach to examine the stakeholder interactions in Romania. A whole network approach requires the definition of network boundaries by establishing a list of relevant stakeholders; and the collection of responses from all stakeholders of the network about each other [25]. We defined a stakeholder as an organization who is involved and influences or is influenced by the Pontocaspian biodiversity research and conservation activities [17, 20]. Based on this definition we initially identified 23 stakeholder institutes in Romania through online research and consultations with partners in the PRIDE project. After engagement, stakeholders which were found to lack any activity or interest in (conservation of) Pontocaspian biodiversity were omitted, resulting in a final list of 17 institutes (Fig 1 and Table 1). We assigned these stakeholders to three different categories based on their function and responsibilities, knowingly academic (Acad), governmental (Gov) and nongovernmental organizations (NGO). For comparison, the Ukrainian network consisted of 22 stakeholders of which nine were academic institutions, five governmental organizations, three nongovernmental organizations and five protected areas (Pa) [17].

**Table 1:** List of the 17 selected stakeholders from Romania divided into three stakeholder categories.

ID	Abbreviation	Category	Organization name	Department/Service
1	CMSN	Acad	CMSN—Museum of Natural Sciences, Constanța	Delfinariu, Constanta
2	GAM	Acad	Grigore Antipa National Museum of Natural History	
3	GEcM	Acad	Constanta Branch of the National Institute for Research and Development on Marine Geology and Geo-ecology–GeoEcoMar	
4	IBB	Acad	Institute of Biology Bucharest, Romanian Academy	Department of Microbiology
5	OUC	Acad	Ovidius University of Constanta	The Faculty of Natural and Agricultural Sciences
6	DDNI	Acad	The Danube Delta National Institute for Research and Development	Biodiversity Conservation and Sustainable use of Natural Resources
7	NIMR	Acad	The National Institute of Marine Research and Development "Grigore Antipa”	
8	UB	Acad	University of Bucharest	Department of Paleontology
9	AZS	Acad	Marine Biological Station of Agigea	
10	DDA	Gov	Danube Delta Biosphere Reserve Authority	
11	LAC †	Gov	Local Environmental Protection Agency in Constanta	
12	ANPA †	Gov	Ministry of Agriculture and Rural Development of Romania	National Agency for Fisheries and Aquaculture
13	MOE	Gov	Ministry of Environment of Romania	Biodiversity Directorate
14	MWF	Gov	Ministry of Waters and Forests	Department for Water, Forests and Fishery
15	MN	NGO	ONG Mare Nostrum	
16	OC	NGO	SEOPMM Oceanic Club	
17	WWF	NGO	WWF Romania	

† Institutions that could not be interviewed for which relationships were imputed

The Danube Delta Biosphere Reserve Authority (DDA) administers the biosphere reserve and serves as a local environmental agency. Besides the administration, it has educational and regulatory (e.g. issuing research permits) functions within the biosphere reserve. The analogous organization in Ukraine, the Danube Biosphere Reserve (DBR) does not have administrative and regulatory functions but instead focuses on research, environmental monitoring and education, as well as on ecotourism. DDA was under commission of the Ministry of Environment of Romania until July 2017, but was transferred under commission of the Romanian Government one week before the interview (July 2017). Presently, DDA is again back under commission of the Ministry of Environment. During the interview, DDA identified itself as a governmental organization and was therefore grouped with governmental organizations.

### Data collection

We obtained the qualitative and quantitative network data using an identical survey questionnaire that was previously used in a similar study in Ukraine [17]. We interviewed the staff members of the institutions or relevant departments during July 2017. Interviews with staff members were undertaken with the knowledge and consent of the organizations to which the staff members were affiliated. Persons that were selected for the interview were all in a central position in the organization and thus aware of most, if not all, organizational aspects relevant to the network analysis. Each stakeholder organization was interviewed about each other organization from the list (Table 1) using the same questions. We extracted the meaning and content of interactions from the interviews and no prior data was used.

We compiled data on the context and the content of interactions among the stakeholders using the question asking interviewees to describe their professional relationships. Next, we asked the interviewees whether the described professional link involved or was related to Pontocaspian (PC) biodiversity. We were mainly interested in PC biodiversity conservation related information, so when the reported interaction between stakeholders was not related to PC biota, we refrained from posing subsequent questions and continued with the next stakeholder from the list (Table 1). Once a PC biodiversity related link was established, the interviewee was asked whether s/he considered the existing relationship sufficient or insufficient to achieve desired levels of collaboration and for what reasons.

We collected the SNA data asking the interviewees to rank the reported PC biodiversity related links based on the frequency of interaction [17]. We used frequency of contact as a measure of strength (weight) of the relationship (see [42, 47]). We defined five weight categories ranging from no contact to very frequent contact (0–4) and integrated the strength definitions as a table in the questionnaire to provide reference for the interviewees. Answers to the questions allowed the generation of directed, weighted, values of information and knowledge transfer in the network.

### Analysis

#### Social network analysis

For readability, we provide the full SNA methodology and term definitions in S1 Text. We translated the collected interviews into an adjacency matrix, a square matrix reporting weights (strength) of all the relational ties (see S1 Dataset). We considered only confirmed information sharing links i.e. relational links described by both stakeholders involved. Unconfirmed links (16% of all the reported relationships) were considered unreliable and were omitted from the study. Tie-strength values of confirmed relationships between pairs of stakeholders did not always match. In case of bi-directional relationship, tie values were left as reported by the stakeholders. In case of unidirectional confirmed links, we selected the lowest and therefore most conservative tie values. Two institutions could not be interviewed resulting in some missing network data. We imputed the missing data using the imputation-by-reconstruction method [48]. We visualized the sociogram using the CRAN R package 'igraph' [49].

The basic network statistics including number of actors and relational ties, graph density and centralization index were calculated using the CRAN R package ‘igraph’ [49]. The mean shortest distance was calculated using the CRAN R package ‘tnet’ [50] because the ‘igraph’ package does not take edge weights into account when measuring the shortest distance. We used frequency of contact as a measure of strength of the relationship and defined strong relationships as the weights ≥3 on a scale ranging from no contact to very frequent contact (S1 Appendix).

Centrality of individual nodes was calculated using degree centrality and betweenness centrality values. We calculated node-level statistics using the CRAN R package ‘tnet’ [50] which considers tie weights and corrects for the number of intermediary nodes. Central stakeholders were regarded as those with centrality scores higher than, or equal to the third quartile threshold values [28, 47, 51].

Brokerage was measured by combining quantitative and qualitative approaches. Brokers are nodes which are between other nodes in a network and have the power to control the flow of information [39, 52, 53]. Quantitatively, brokerage was measured through betweenness centrality and Burt’s constraint metrics [39, 52]. Qualitatively, we examined the network narratives and extracted evidence that stakeholders are actually engaging in brokering behavior, such as mobilization of information, deliberation between different types of stakeholders and mediating between working groups to address conservation issues [54]. Here, we regarded stakeholders as brokers when they had high betweenness scores, low Burt’s constraint values, and were engaged in brokering behavior. We used only the strong ties (≥ 3) to calculate betweenness centrality and Burt’s constraint metrics as these reflect regular contacts. We calculated Burt’s constraint utilizing CRAN R package ‘igraph’ [49].

Finally, we used a null-model test to identify the presence of ‘network homophily’ in the network. ‘Network homophily’ is the selective linking between actors based on specific attributes, in our case the category of stakeholder institutes [55]. With a null-model test, we tested whether densities within and between stakeholder groups (defined by the stakeholder category) were significantly higher or lower than random expectation.

#### Qualitative analysis

We used the ‘inductive approach’ for qualitative analysis, so the themes (recurrent unifying concepts or statements about the content/subject of the inquiry) of interaction and perceived sufficiency of interaction were determined based on the collected data and not on prior knowledge or assumptions [56, 57]. The themes were established from the collected interviews based on repetitions [58]. We used a ‘constant comparison’ method to refine the dimensions of established themes and to identify the new themes [59]. We then counted the identified themes and determined their relative importance based on the order of frequency. We grouped the identified themes of interaction based on similarity in two categories, knowingly ‘collaboration relations’–links between the stakeholders consisting of joint action, and ‘communication relations’–links between the stakeholders mostly used for conveying information.

#### Ethics statement

The social network analysis of stakeholder organizations which we conducted here is not subject to ethical screening as for example is required for medical and/or socio-medical studies, which involve personal data. As such, we did not conduct a priori ethics review nor is there any established procedure within our organization (Naturalis Biodiversity Center) which could be followed. We informed all participants prior to the interviews that they were being interviewed on behalf of the organization which they represent, and that the results would be part of a publication. We assured all participants that they would not be individually identifiable and asked for their consent.

## Results and discussion

Conservation of Pontocaspian (PC) biodiversity is critically dependent on adequacy of conservation measures and coordination of actions across their distribution range—the northern part of the Black Sea and the Caspian Sea region. This paper assesses the adequacy of stakeholder networks for conservation in two countries covering a large part of the native range of PC biota. We compare the social network structures of stakeholders involved in biodiversity conservation in Romania and Ukraine, based on new data from the former and data from a previous published paper from the latter [17]. Then we discuss the implications of the Romanian results for effective conservation and compare these to the findings from Ukraine. We examine the challenges within, as well as beyond the network structure for optimal PC biodiversity conservation and provide recommendations for improved cross-border conservation efforts.

### Network structure

**Fig 2:**
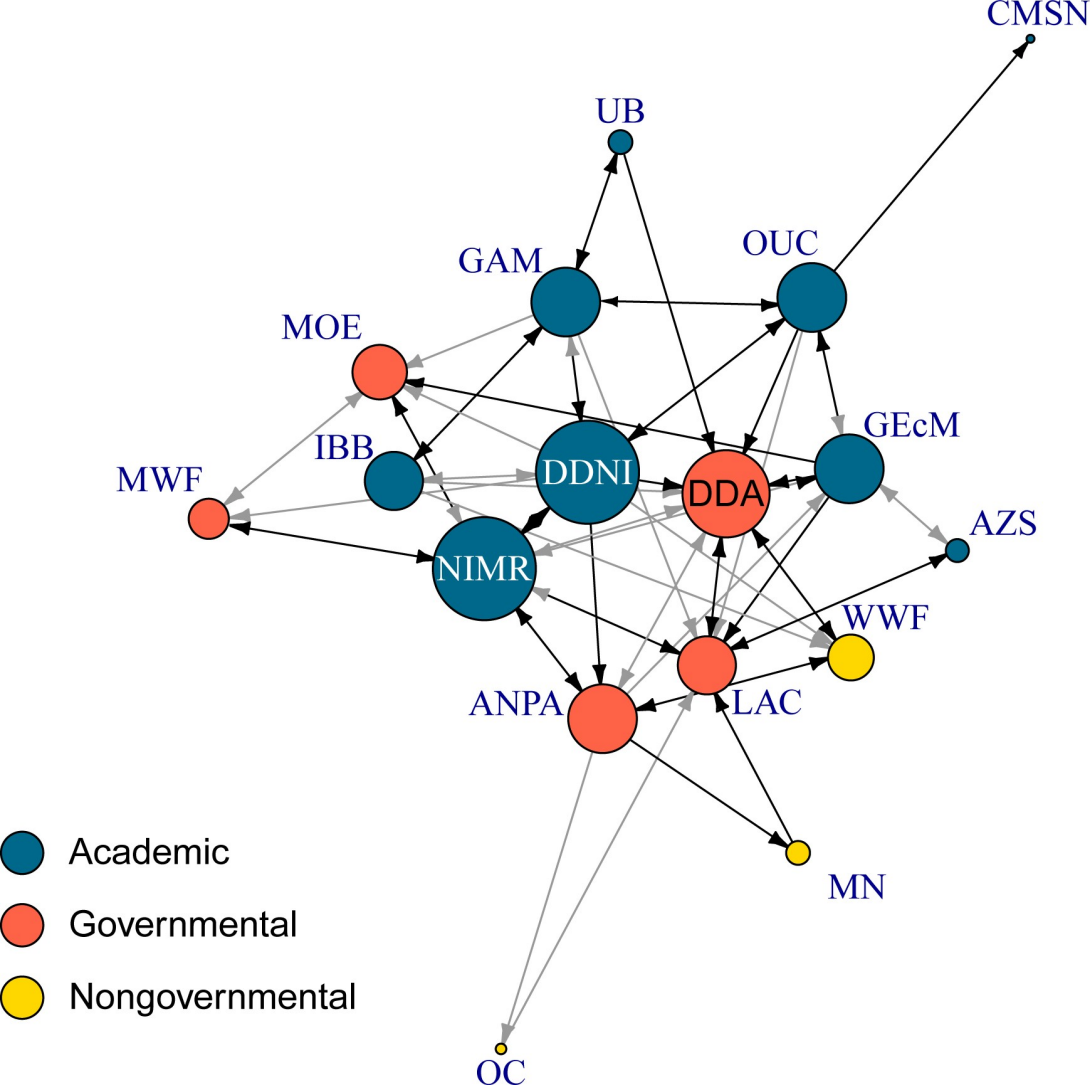
Sociogram of Romanian stakeholders involved in Pontocaspian biodiversity conservation and conservation planning. Nodes represent organizations (see Table 1 for institution acronyms). The size of the nodes corresponds to the node strength (sum of weights of all its links). Arrows represent relationships between the nodes. Black arrows represent strong relationships (value ≥3). Gray arrows represent weak relationships (value < 3).

The Romanian network was smaller compared to Ukrainian one (17 vs. 22 stakeholders respectively) and also less connected. In Romania, 15 out of the 17 stakeholder institutions were interviewed (covering 88% of the network data). Fourteen organizations were interviewed through face to face in-depth interviews and one organization through an electronic questionnaire via email. The remaining two institutions could not be reached and data were imputed (Table 1). The studied network in Romania was not well connected (Fig 2) with a total number of 63 relational ties out of 272 potential ties, resulting in a network edge density measure of 23% (Table 2). For comparison, the Ukrainian network had an edge density value of 41%. On average each organization in Romania had 7 relational ties with other stakeholders in the network, while in Ukraine each stakeholder had on average 17 ties. This resulted in larger mean distance between stakeholders in the Romanian network compared to Ukrainian one (2.2 vs 1.5 respectively). The Romanian network had a lower degree of centralization score (20%) than the Ukrainian network (38%), meaning that the former was less centralized than the latter. The correlation of incoming and outgoing ties, although positive in both networks, was lower in Romania compared to Ukraine (rho = 0.38 in Romania vs. rho = 0.78 in Ukraine) indicating that information exchange was in general less reciprocated in Romania (Table 2). When governmental organizations (including the DDA) were omitted from the Romanian network, the correlation increased (rho = 0.79), suggesting that the governmental organizations in Romania received information from multiple sources but did not share similarly. In both countries, the majority of relationships were strong (59% in Romania and 61% in Ukraine), indicating regular interactions.

**Table 2:** Network statistics for Romanian stakeholder network compared to the previously published Ukrainian stakeholder network (in grey) [17].

Network data	Romania	Ukraine
Total actors	17	22
Total No. of ties	63	191
Mean degree	7	17
Density (%)	23	41
Degree of centralization (%)	20	38
Tie reciprocity (rho)	0.38	0.78
Tie reciprocity (rho) excluding the Gov. organizations	0.79	0.76
Strong/weak ties (%)	59/41	61/39
Mean shortest distance	2.2	1.5

### Network relations

**Fig 3:**
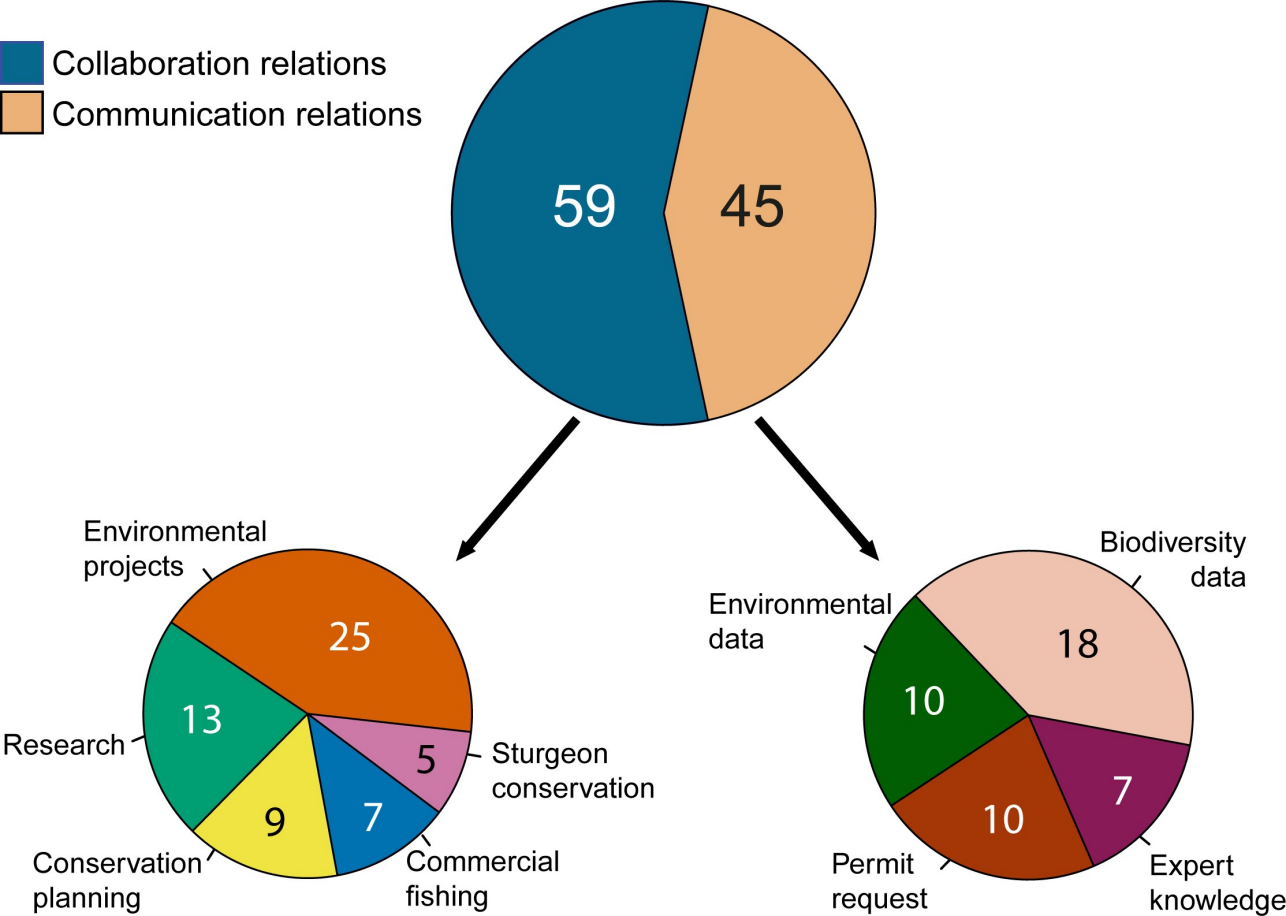
Frequencies of interaction themes among the stakeholder organizations. Values in the pie charts represent absolute number of times each theme was mentioned. See theme definitions in S2 Table.

Unlike in Ukraine, the majority of interactions among stakeholder organizations in Romania consisted of ‘collaboration relations’ while transfer of information was less common (Fig 3 and S1 Table). Interactions in Romania were mostly achieved through joint projects. For example, the collaboration themes ‘environmental projects’, ‘sturgeon conservation’ and ‘conservation planning’ were all based on common projects (S1 Table). Within these projects, exchange of relevant information and data was easily achieved, as indicated by the interviewees. Outside projects, however exchange of comprehensive data in Romania was either not possible or was subject to payment. Thirty-two relational links in the network were represented by a single theme of interaction. Twenty-three links had 2 themes of interaction, seven links had 3 themes of interaction and 1 link had 5 themes of interaction. Similar to Ukraine, links represented with more themes were significantly stronger than links represented with less themes (S1 Fig).

In Romania, like in Ukraine, Pontocaspian species played a minor and mostly incidental role in inter-organizational relations (Fig 3 and S1 Table), indicating low priority for PC biodiversity conservation. Collaborative interactions theme ‘conservation planning’ involved biodiversity monitoring according to the EU Habitats Directive (Article 17), and planning of conservation activities within Natura 2000 sites, coinciding with PC habitats (e.g. Razim-Sinoe Lake Complex as a Natura 2000 site https://natura2000.eea.europa.eu/?query=Natura2000Sites_9883_1, Site Code: ROSPA0031). Furthermore, the theme ‘Research’ involved joint fieldwork and publications on the biodiversity of the Black Sea coastal areas, lagoons, rivers and lakes, which also cover the PC habitats. Interactions within the ‘commercial fishing’ theme involved some PC fish species such as the Pontic shad and some invasive species, such as the veined Rapa whelk, which is potentially harmful to native PC species. Similar to Ukraine, ‘sturgeon conservation’ was the only collaborative theme, which directly targeted PC biodiversity conservation. This theme, however, primarily focused on sturgeon species and other PC groups were left out. Communication relations mostly included a) information transfer related to reporting obligations to the EU (Fig 3 and S1 Table; themes ‘biodiversity data’ and ‘environmental data’), b) administrative work to implement the research projects (theme ‘permit request’) and c) sharing of project management experiences and advice; all of which occasionally covered the PC habitats. This is indicative of low priority for PC biodiversity conservation on both the national and European agendas, with the notable exception of sturgeon species [60]. Individual scientific organizations, such as Grigore Antipa National Museum of Natural History, Constanta Branch of the National Institute for Research and Development on Marine Geology and Geo-ecology–GeoEcoMar, and the Danube Delta Research Institute did possess PC species occurrence and distribution data, but they reported that this data is not utilized because governmental organizations and NGOs file no data requests (Tables 4 and S1).

### Perceived sufficiency of network relations

A total of 19 relational ties (44% of 43 ties for which sufficiency was indicated by the interviewed stakeholders) were reported to be insufficient in Romania to achieve the desired levels of collaboration and information exchange (S2 Table). We identified 3 themes of insufficient interactions–‘lack of funding’, ‘political constraints’ and ‘institutional turnover’. For comparison, in Ukraine 31% of relational links were construed as insufficient. The causes for insufficient relationships were different in two countries. ‘Lack of funding’ in Romania (mentioned 10 times), and ‘budget constraints’ in Ukraine (mentioned 18 times) were the most prominent factors limiting collaboration. Besides the general lack of funding available for research and conservation, which was a common characteristic of both themes, ‘budget constraints’ also referred to unfavorable funding schemes in Ukraine which restricted the participation of different stakeholder categories in a project [17]. However, ‘budget constraints’ did not have effect on exchange of information in Ukraine, while ‘lack of funding’ in Romania affected the access to biodiversity and environmental information (see S2 Table). Besides publicly funded projects in Romania, the EU LIFE Program is the major source for conservation funding [61]. An earlier study on collaboration networks across Europe found that once a project was awarded to an organization in Romania, such organization became less prone to collaborate with other organizations in other projects, so project management experiences were not shared among stakeholders [62]. This was attributed to difficulties in the implementation of EU LIFE projects [62]. Additionally, according to our findings the reduced collaboration occurred also due to institutional competition among stakeholders which encouraged organizations to keep data to themselves as a competitive advantage to attract future grants (see S2 Table; theme ‘lack of funding’).

‘Political constraints’ (mentioned 6 times) and ‘institutional turnover’ (mentioned 3 times) were reported only in Romania and not in Ukraine. Continuous institutional rearrangements were found to complicate firstly the establishment and secondly the maintenance of relationships in Romania (S2 Table; theme ‘institutional turnover’), resulting in low network density (Table 2). For example, the Ministry of Environment reported an absence of relationship with DDA (Fig 2), and described the situation as follows: “DDA used to be under our structure until recently, but they are now coordinated by the government and we do not know how the new dialog will be because we are currently in a process of rearrangements”. Institutional turnover also resulted in many unconfirmed relations. For example, out of 7 outgoing ties from the Marine Biological Station of Agigea (AZS) 5 were not confirmed (S1 Dataset) as AZS was still deemed to be part of the University of Iasi and not yet recognized as an independent organization by many of the stakeholders. This finding corroborates an earlier study which suggested that continuous institutional reforms of the public sector is a result of adjustments to the EU institutional structures which does not always have positive outcomes in Romania [15]. According to the same study, however, continued reforms of public sector are necessary to ensure access to national funds for scientific research [15]. Therefore, institutional turnover may be expected to persist in the coming years in Romania.

Unlike in Ukraine, the involvement of governmental organizations in the studied network was limited by bureaucratic barriers (S2 Table; theme ‘political constraints’), which resulted in few reciprocated ties between governance actors and other stakeholder categories (Table 2). Lack of reciprocated communication (governmental stakeholders receiving information from multiple sources but not sharing back to the network) is indicative of a strong hierarchy in conservation governance [63]. According to literature, stakeholder engagement in conservation planning is often interpreted by the governmental organizations in Romania as intersectoral cooperation and engagement, which results in seeking collaboration with other governmental organizations and international actors rather than in collaboration with local organizations and NGOs, resulting in hierarchical governance systems [16, 64, 65]. However, the theme ‘legal limitations’ which in Ukraine mostly referred to contradicting national laws and uncoordinated actions of regional administrations [17], was not mentioned in Romania, indicating higher consistency in conservation policies in Romania. In both countries most of the insufficient relationships were represented by strong links, suggesting that frequent interactions were not a guarantee for effective collaboration (see S2 Table).

### Stakeholder centrality and brokerage

In Romania five central stakeholders were identified based on their degree centrality scores (Table 3), compared to six in Ukraine [17]. In both networks three out of nine academic institutions had a degree centrality score higher than or equal to the third quartile threshold value (≥11 in Romania and ≥20 in Ukraine), indicating high involvement of these organizations in the exchange of relevant information. Unlike in Ukraine, where the major decision-making organization (Ministry of Ecology) was the most central stakeholder, in Romania, the analogous institution (Ministry of Environment) was not actively involved in the network. Instead, the Local Environmental Protection Agency in Constanta (LAC) was the central governmental institution with high degree centrality score. The Danube Delta Biosphere Reserve Authority (DDA) in Romania and the Danube Biosphere Reserve Administration (DBR) in Ukraine were both active in stakeholder networks with high degree centrality scores. Nongovernmental organizations had few connections in both countries. All the central stakeholders in Ukraine and Romania had more strong than weak connections.

**Table 3:** Node-specific centrality measures and interaction categories from Romania.

Abbr.	Degree centrality	No. ties Strong/weak	Betweenness centrality	Burt’s constraint	Collaboration relations	Communication Relations
DDNI	**13 (4, 9)**	7/6	**57**	36	15 (6, 9)	14 (3, 11)
NIMR	**13 (6, 7)**	9/4	**89**	**25**	16 (8, 8)	4 (1, 3)
DDA	**12 (9, 3)**	8/4	**54**	**25**	10 (6, 4)	14 (13, 1)
GAM	**11 (5, 6)**	7/4	45	**32**	13 (4, 9)	8 (3, 5)
LAC †	**11 (8, 3)**	7/4	39	**26**	6 (4, 2)	11 (9, 2)
GEcM	10 (4, 6)	7/3	20	36	8 (4, 4)	8 (0, 8)
ANPA †	10 (4, 6)	6/4	**64**	36	9 (3, 6)	2 (2, 0)
OUC	9 (3, 6)	7/2	48	**32**	8 (5, 3)	6 (2, 4)
MOE	8 (5, 3)	2/6	0	66	8 (4, 4)	4 (4, 0)
IBB	6 (2, 4)	2/4	0	100	6 (3, 3)	6 (2, 4)
WWF	6 (4, 2)	4/2	**49**	50	7 (4, 3)	4 (3, 1)
MWF	5 (3, 2)	2/3	0	100	4 (2, 2)	1 (1, 0)
AZS	4 (2, 2)	2/2	0	100	4 (2, 2)	2 (1, 1)
UB	3 (1, 2)	3/0	0	56	2 (2, 0)	2 (0, 2)
MN	2 (1, 1)	2/0	0	50	1 (1, 0)	1, (0,1)
OC	2 (1, 1)	0/2	0	NA	1 (1, 0)	1, (0,1)
CMSN	1 (1, 0)	1/0	0	100	0	1 (1, 0)

Values between brackets under the ‘Degree centrality’ represent the in-degree and out-degree measures respectively. In bold are values higher than, or equal to the third quartile threshold (lower or equal to the first quartile threshold in case of ‘Burt’s constraint’). Burt’s constraint value for OC is not defined (NA) as the calculation was based only on strong ties (≥ 3).

† Institutions that could not be interviewed for which relationships were imputed

Two out of six central stakeholders in Romania, namely the National Institute of Marine Research and Development "Grigore Antipa” (NIMR), and the Danube Delta Biosphere Reserve Authority (DDA) had a structurally favorable position to act as brokers based on betweenness centrality and Burt’s constraint scores (Table 3). Qualitative data, however, showed that these structurally well-positioned organizations were not engaging in brokering behavior with regard to Pontocaspian biodiversity. From network narratives we found that NIMR was a national focal point in many international bodies, such as UNESCO, the Black Sea Commission and GEF/Black Sea, among others, and very actively involved in the Black Sea Biodiversity conservation. However, its primary focus was on Marine and not on Pontocaspian biodiversity conservation (S3 Table). In the studied network NIMR was collaborating with other organizations, e.g. with the Ministry of Environment, Danube Delta National Institute for Research and Development and DDA on conservation planning in Natura 2000 sites, which sometimes incidentally involved PC habitats. But it did not have any incentive to initiate PC biodiversity relevant conservation actions, either due to low priority for PC biodiversity conservation or lack of knowledge on PC species. The second structurally well positioned organization to act as broker was DDA. This organization was a major local administrative body and was found to mostly request and receive information from other stakeholders but rarely communicated the knowledge back to the network (Tables 2 and 3 and S3). From the narratives we learned that this organization was experiencing frequent institutional turnover and was politically constrained (see S2 Table), which complicated the establishment of relationships. As a result, DDA was not found to facilitate any brokering behavior and served as a local protected area administrator and a data aggregator (Table 3).

WWF accounted for high betweenness values in both networks; however, they did not directly bridge many disconnected nodes (indicated by their high Burt’s constraint scores). The qualitative data showed that WWF Romania and WWF Ukraine were actively involved in the conservation of sturgeon species (S3 Table) through the enforcement of conservation laws and awareness raising [17]. They had large number of volunteers in both countries and sometimes brought the otherwise disconnected stakeholder organizations together for joint conservation action. Their work, however, mostly focused on charismatic PC species and the wider PC taxa was absent from their conservation agenda.

### Stakeholder group connectivity

Across the Romanian network, different stakeholder categories had various tie densities, but connectedness was not significantly higher than random expectation indicating the absence of network homophily (Table 4). In Ukraine, strongly connected academic institutions were found with a significantly higher within group density value than expected by chance suggesting high levels of connectedness within this group [17]. Most relations among stakeholder categories in Romania were collaboration relations, with the exception of links among academic and governmental organizations, which mostly consisted of knowledge transfer (Table 4). When in contact, academic institutions requested research permits from governmental organizations and reported on study results (theme ‘permit request’). Additionally, governmental organizations were found to regularly request environmental and biodiversity data from academic organizations for reporting to the EU and international treaties (themes ‘biodiversity data’ and ‘environmental data’). Some of the links among these stakeholder groups were insufficient due to political constraints, institutional turnover, and/or lack of funding (Table 4).

**Table 4:** Stakeholder group relations.

Category (No. ties)	Density (%)	No. ties strong/ weak	Insufficient interactions (No. mentioning)	Collaboration relations (No. mentioning)	Communication relations (No. mentioning)
Gov-Gov (6)	30	2/4	NA	Conservation planning (4)	Environmental data (2)
				Commercial fishing (2)	
Acad-Acad (21)	29	14/7	Lack of funding (7)	Projects (14)	Biodiversity data (12)
				Research (13)	
NGO-NGO (0)	0*	NA	NA	NA	NA
Gov-NGO (8)	14	6/2	Political constraint (2)	Sturgeon conservation (4)	Expert knowledge (2)
				Projects (2)	Environmental data (1)
				Commercial fishing (2)	
Acad-Gov (26)	14	15/11	Political constraint (4)	Projects (9)	Permit request (10)
			Institutional turnover (3)	Conservation planning (5)	Biodiversity data (6)
			Lack of funding (2)	Commercial fishing (3)	Environmental data (6)
					Expert knowledge (3)
Acad-NGO (2)	1.5*	0/2	Lack of funding (1)	Sturgeon conservation (1)	Expert knowledge (2)
					Environmental data (1)

Values between brackets under ‘Category (No. ties)’ represent the number of existing relational ties in Romania within and between stakeholder groups.

An * indicates significant difference from random expectation (*p* < 0.05) according to the null-model test.

Nongovernmental organizations were marginally involved in both Romanian and Ukrainian networks. In Romania, NGOs were significantly less connected to the academic institutions than expected by chance and had no PC biodiversity related links among themselves (Table 4). In Ukraine, NGOs were also significantly less connected to academic organizations and had only two PC biodiversity related links among themselves [17]. Marginal involvement of NGOs in Romania has been observed in a previous study in the broader conservation context of the Natura 2000 governance network [66], indicating that our findings may not be unique to PC biodiversity conservation network. Effective biodiversity conservation requires information exchange between diverse stakeholder categories [37, 42], which awards greater stakeholder ownership to conservation outcomes and ensures equal spreading of the costs and risks of conservation actions [67]. Therefore, more interaction between NGOs and other stakeholders will likely benefit conservation of PC biodiversity.

### Conservation implications of the Romanian vs. Ukrainian networks

According to network theory [35, 41, 54] the observed landscape of stakeholder interactions in Romania is structurally suboptimal–it is decentralized, has few and unreciprocated ties, and few structurally well positioned stakeholder organizations which lack incentives to utilize their favorable positions to initiate PC biodiversity related actions (Fig 2 and Tables 2 and 3). Decentralized networks are suitable for long-term environmental planning and complex problem solving, as a result of stakeholders across multiple disciplines contributing to the solution of a problem [35]. A centralized network with one or few very central stakeholders, however, usually is more effective in the initial phase of the conservation process when resources need mobilization and the central coordination of joint actions is required [35, 43]. While social and political setting in Romania and Ukraine to deal with biodiversity conservation issues are different, in terms of PC biodiversity conservation it can be argued that the two countries are in a similar, initial phase. In both countries PC biodiversity is recognized to be threatened and partly included in legal documents [e.g. see 68–70], but is not yet included in conservation planning processes and implementation as it is absent from collaboration relations between relevant stakeholders in both countries (Fig 3 and Tables 4 and S1). If supplied with knowledge on PC biodiversity and the right incentives, in the initial phase of conservation a well-connected, centralized network in Ukraine is better placed to translate knowledge into effective conservation actions [17] through engaging the central, powerful stakeholders [35, 43]. The Romanian network on the other hand in its current stage is less suited to facilitate improvements as it is decentralized with marginal involvement of governance actors and NGOs (Tables 2 and 4).

Besides the lack of knowledge on PC biodiversity and the incentives to initiate conservation actions, the stakeholder networks in both countries are challenged by the additional social variables, most notably the limited available funding for biodiversity conservation (S2 Table). In Romania collaboration stopped when the funding period was finished and projects were concluded. In Ukraine, organizations continued to collaborate and exchange information beyond the duration of projects [17]. Romanian stakeholders were involved in many more projects than Ukrainian stakeholders (Fig 3 and S1 Table), and many of these projects were EU funded [62]. Yet, the Romanian network was less dense than the Ukrainian one due to the difficulty of implementing EU projects, which prevented organizations awarded an EU project to participate in other projects [62], resulting in a low network density (Fig 2 and Table 2). Similarly, the authoritative state governance system was more consequential for PC biodiversity conservation in Romania (S2 Table; theme ‘political constraints’) than in Ukraine [17], resulting in lack of collaboration between governance actors and other stakeholder categories in Romania (Tables 2 and 4). Contrary to our findings, it was suggested that the accession to the EU has played a major role in transposing the environmental governance and biodiversity conservation practices towards more collaborative, inclusive system in Romania [16]. However, challenges remain, which are suggested to be caused by lack of previous experience with the participatory conservation practices [16]. Consequently, improvements can be expected in Romania as the collaborative system of conservation matures. Importantly, while in Ukraine contradicting national laws and uncoordinated actions of regional administrations were common [17], they were not the case in Romania; indicating higher consistency in conservation policies in Romania, which in turn may be the result of the accession to the EU Acquis.

### Coordinating joint Pontocaspian biodiversity conservation actions

Romania and Ukraine share the Danube Delta, the Black Sea coastline and associated habitats in which Pontocaspian biota occurs (Fig 1), which may benefit from a coordinated action of both countries [71]. Some of the PC species, e.g. the sturgeon species, are mobile and not limited to the administrative and political boundaries [72]. Furthermore, PC species have a patchy distribution in Ukraine and Romania and face similar pressures in both countries [9, 18, 19]. Cross-border collaboration is therefore instrumental to achieve common conservation objectives and optimal conservation action [71, 73]. Sharing the management experiences and best practices among the organizations from both countries can help to the development of common organizational awareness and embolden joint efforts and understanding [73, 74].

The great significance of cross-border collaboration has been recognized by international conventions and the EU, which resulted in several collaborative projects [75]. In our interviews we did not specifically address cross-border collaboration between Romania and Ukraine with regard to PC biodiversity, but from the network narratives we learned that institutions in both countries are aware of each other and some collaboration exists. Established programs relevant to PC biodiversity conservation are the cross-border cooperation program (within the European Neighborhood Instrument - https://www.euneighbours.eu/en) and the EU LIFE program. The former includes the “Black Sea”, “Danube”, and other bilateral or trilateral (including Moldova) ecological programs with substantial budgets. Usually in their formulations the term "Pontocaspian" does not exist, but these projects mainly concern the habitats of PC fauna (Danube Delta and Prut River, Lower Dniester and the Black Sea coastline of Ukraine, Romania and Bulgaria). The EU LIFE program targets Danube sturgeons. For other PC taxa we did not find evidence for deep collaboration. The PRIDE project (http://www.pontocaspian.eu/) was a pioneering EU funded project, which, in collaboration with WWF Ukraine, attempted to integrate the entire PC community in the sturgeon related awareness raising activities for different coastal protected area administrations and local residents in Ukraine. Future projects that can extend the current organizational focus from flagship species to the entire PC biota in Ukraine and Romania are critically important. Such projects can be expected to raise awareness of the need of PC biodiversity conservation and increase the interest of governmental and nongovernmental organizations to collaborate more and exchange the relevant information.

## Conclusions

We found structurally different networks of stakeholder organizations in Romania and Ukraine. However, PC biodiversity was not a driver of inter-organizational relations in either of the countries, resulting in incidental coverage of this biota in conservation practices. In an earlier study from Ukraine, we concluded that the maintenance of existing network is a necessary base, and can be expected to result in increased conservation action if the content of interactions is improved and funding and legal limitations are resolved. In Romania, such social variables are more consequential for the network functioning resulting in a hierarchical, non-inclusive system of conservation planning, continuous institutional reforms, and reduced collaboration. Improvements can be expected, however, as the adjustments to the EU institutional structures and the participatory conservation governance systems mature in Romania. Fostering cross-border collaboration through new calls for project proposals from the state and the EU budgets, which involve wider Pontocaspian taxa, will likely increase the PC conservation awareness and interest of different types of stakeholders in both countries to engage more in the conservation actions related to PC biota. Extending the Sturgeon networks to the other, non-charismatic Pontocaspian species may be a preferable course to initiate such action.

## Supporting information

S1 Dataset Raw data used for performing the network analyses. (XLSX)

S1 Fig Number of themes representing a relational link and the strength of the link. (TIF)

S1 Table Identified themes of stakeholder interactions and their descriptions. (DOCX)

S2 Table Identified themes of insufficient interaction and their description. (DOCX)

S3 Table Number of mentioning of interaction themes by individual stakeholders. (DOCX)

S1 Text Social network analysis methods. (DOCX)

S1 Appendix Survey questions. (PDF)
