# Supplementary figures and images for: Social network analysis and the implications for Pontocaspian biodiversity conservation in Romania and Ukraine: A comparative study
Source: PLoS One. 2020 Oct 23;15(10):e0221833. doi: 10.1371/journal.pone.0221833 (PMC7584225; doi:10.1371/journal.pone.0221833)

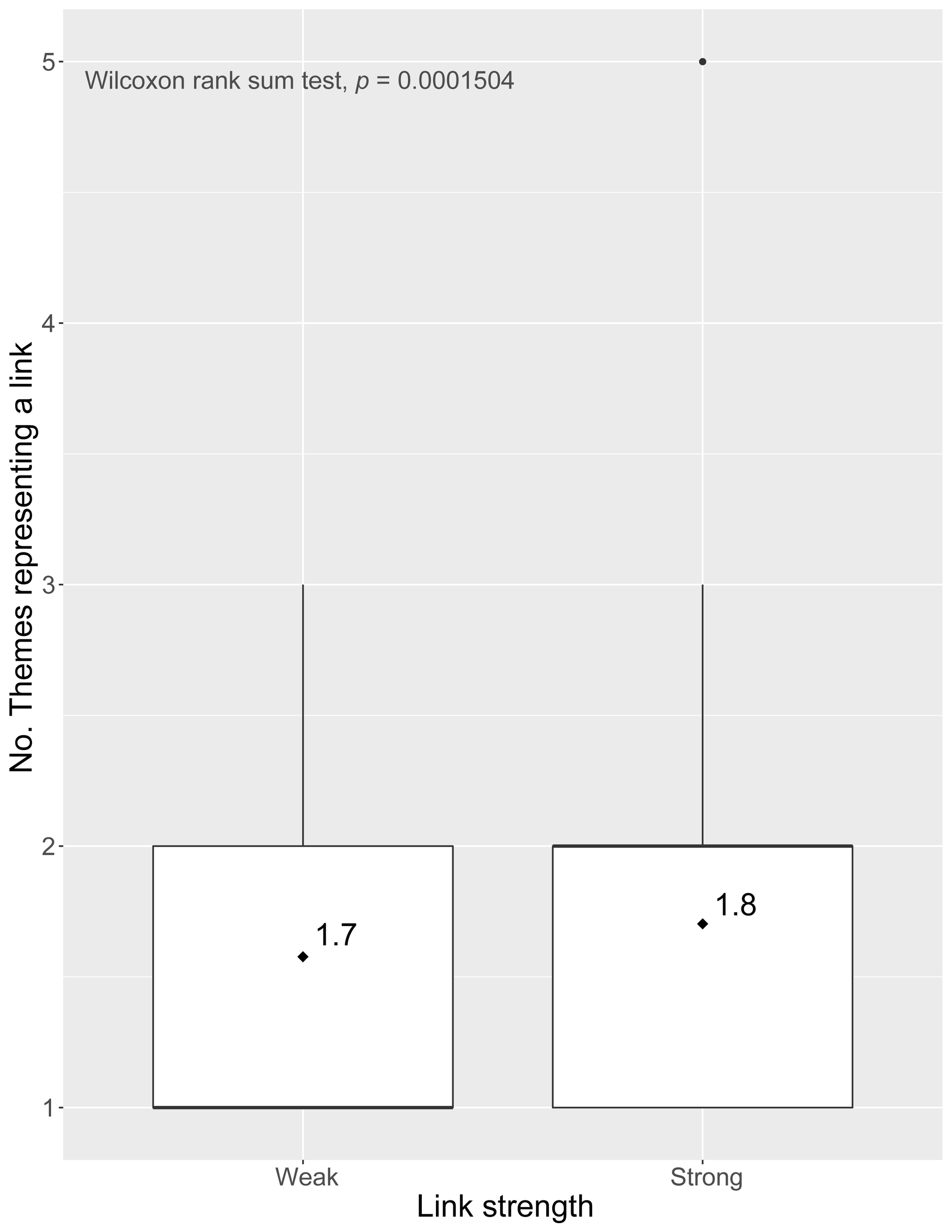

Supplement: S1 Fig — (TIF) [file pone.0221833.s002.tif]
