# Supplementary material for: Social network analysis and the implications for Pontocaspian biodiversity conservation in Romania and Ukraine: A comparative study
Source: PLoS One. 2020 Oct 23;15(10):e0221833. doi: 10.1371/journal.pone.0221833 (PMC7584225; doi:10.1371/journal.pone.0221833)
Supplement: S1 Table — (DOCX) [file pone.0221833.s003.docx]

**S1 Table. Identified themes of stakeholder interactions and their descriptions.** ‘Frequency’ reports the number of mentioning of identified themes by the interviewees. Numbers between brackets reflect how many times the theme was associated to strong vs. weak relational links.

| Category | Theme name | Theme description | Frequency (strong/weak) |
| --- | --- | --- | --- |
| Collaboration relations | Environmental projects | Stakeholders are partners in joint environmental projects, which involve PC habitats, and exchange information according to the project needs. Projects mentioned by the stakeholders were e.g. EU LIFE projects on implementing EU Habitats Directive through generating knowledge for Article 17 reporting; projects on establishing limits on organic pollutants. | 25 (16/9) |
|  | Research | Stakeholders conduct joint fieldworks and publish scientific papers on biodiversity of the Black Sea coastal lagoons, lakes and rivers, which sometimes involve Pontocaspian species and habitats. | 13 (6/7) |
|  | Conservation planning | Stakeholders co-manage protected areas and Natura 2000 sites, plan conservation activities within Natura 2000 network of protected areas, provide scientific support for nominating and establishing new protected areas; and jointly develop and implement nature restoration projects. | 9 (6/3) |
|  | Commercial fishing | Stakeholders are involved in joint management and planning of commercial fishing activities, which sometimes concerns Pontocaspian species and habitats, e.g. Pontic shad in Danube Delta. This collaborative theme involves exchange of information on amount of fish catch per year, the vessels, the quotes and the species that are being caught; also, the strategies and policy related to fishing and the control of fishing activities. | 7 (3/4) |
|  | Sturgeon conservation | Stakeholder organizations are partners in sturgeon conservation related projects, for example, the “LIFE for Danube Sturgeons” (<https://danube-sturgeons.org/the-project/>). | 5 (4/1) |
| Communication relations | Biodiversity  data | Stakeholders exchange information related to Pontocaspian habitats and/or species outside the projects. This theme involves requesting the data, mostly by the governmental organizations for reporting to the EU, with or without payment for data. | 18 (12/6) |
|  | Environmental data | Stakeholders exchange information on the state of environmental conditions. For example, information on water quality parameters, silt deposition and evolution of the Black Sea shoreline, hydrological data, informing on facts of transgression from the field, environmental impact assessment results for exploitation of biological resources. | 10 (6/4) |
|  | Permit request | Academic stakeholders apply for permits of research to the governmental organizations and report on generated study results as obliged by the law. Research occasionally includes Pontocaspian species and habitats. | 10 (6/4) |
|  | Expert knowledge | Stakeholders share expert knowledge, advice, recommendations, consultations and experiences on different biodiversity initiatives, ecosystem restoration projects, decisions and investments. Shared knowledge occasionally pertains to Pontocaspian species and habitats. | 7 (4/3) |
