# Supplementary material for: Social network analysis and the implications for Pontocaspian biodiversity conservation in Romania and Ukraine: A comparative study
Source: PLoS One. 2020 Oct 23;15(10):e0221833. doi: 10.1371/journal.pone.0221833 (PMC7584225; doi:10.1371/journal.pone.0221833)
Supplement: S2 Table — (DOCX) [file pone.0221833.s004.docx]

**S2 Table. Identified themes of insufficient interaction and their description.** ‘Frequency’ reports total number of times a theme was mentioned. Values between brackets represent number of times theme characterized strong vs. weak relational links.

| Name | Description | Frequency (strong/weak) |
| --- | --- | --- |
| Lack of funding | Desired levels of collaboration cannot be achieved due to shortage of finances which translates into either of the two scenarios: 1) Organizations are open for collaboration but have no common projects in which to collaborate; or 2) Scientific organizations that hold most biodiversity information (e.g. DDNI and GAM) do not share information for free so the organizations which are in need of information but cannot afford it reported interaction as insufficient. Scientific organizations in Romania are insufficiently funded by the government and data quality, availability and persistence are dependent on their success to find additional funding. | 10 (5/5) |
| Political constraints | Governmental organizations are not open for consultations and collective, joint conservation planning because they are strongly influenced by the politics. Academic and non-governmental organizations express interest in more collaboration and exchange of information with the governmental authorities, while the government does not respond due to different interests or priorities. | 6 (6/0) |
| Institutional turnover | Desired levels of interaction cannot be achieved due to continuous institutional reforms, which result in confusion among the organizations and continuous need for new agreements and dialog on the new format of collaboration frameworks. For example, from the interviews we learned that the Ministry of Environment (MOE) and Ministry of Waters used to be one organization, but were split up shortly before the interview; the Danube Delta Biosphere Reserve Authority (DDA) was transferred from the MOE to central government one week before the interview, but currently operates again under the commission of the MOE; and the Marine Biological Station of Agigea (AZS) became a separate organization 1 year before the interview, previously being a research station of the University of Iasi. | 3 (2/1) |
