# Supplementary material for: Social network analysis and the implications for Pontocaspian biodiversity conservation in Romania and Ukraine: A comparative study
Source: PLoS One. 2020 Oct 23;15(10):e0221833. doi: 10.1371/journal.pone.0221833 (PMC7584225; doi:10.1371/journal.pone.0221833)
Supplement: S3 Table — (DOCX) [file pone.0221833.s005.docx]

|  |  |  | Collaboration relations | | | | | Communication relations | | | |
| --- | --- | --- | --- | --- | --- | --- | --- | --- | --- | --- | --- |
| Abbr. | Legal status | Degree | Environmental projects | Research | Conservation planning | Commercial fishing | Sturgeon conservation | Biodiversity data | Environmental data | Permit request | Expert knowledge |
| DDNI | Acad | 13 (4,9) | 9 (4,5) | 6 (2,4) | 0 | 0 | 0 | 8 (3,5) | 2 (0,2) | 2 (0,2) | 2 (0,2) |
| NIMR | Acad | 13 (6,7) | 9 (4,5) | 2 (2,0) | 3 (1,2) | 2 (1,1) | 0 | 0 | 3 (1,2) | 1 (0,1) | 0 |
| DDA | Gov | 12 (9,3) | 4 (3,1) | 0 | 2 (1,1) | 2 (1,1) | 2 (1,1) | 1 (1,0) | 5 (4,1) | 6 (6,0) | 2 (2,0) |
| GAM | Acad | 11 (5,6) | 9 (3,6) | 4 (1,3) | 0 | 0 | 0 | 6 (3,3) | 0 | 1 (0,1) | 1 (0,1) |
| LAC † | Gov | 11 (8,3) | 1 (1,0) | 0 | 5 (3,2) | 0 | 0 | 1 (1,0) | 6 (4,2) | 3 (3,0) | 1 (1,0) |
| GEcM | Acad | 10 (4,6) | 2 (1,1) | 5 (2,3) | 0 | 1 (1,0) | 0 | 4 (0,4) | 2 (0,2) | 1 (0,1) | 1 (0,1) |
| ANPA † | Gov | 10 (4,6) | 0 | 0 | 0 | 7 (2,5) | 2 (1,1) | 0 | 0 | 1 (1,0) | 1 (1,0) |
| OUC | Acad | 9 (3,6) | 4 (2,2) | 4 (3,1) | 0 | 0 | 0 | 4 (2,2) | 0 | 2 (0,2) | 0 |
| MOE | Gov | 8 (5,3) | 4 (2,2) | 0 | 4 (2,2) | 0 | 0 | 3 (3,0) | 0 | 0 | 1 (1,0) |
| IBB | Acad | 6 (2,4) | 3 (2,1) | 2 (1,1) | 0 | 0 | 1 (0,1) | 4 (2,2) | 0 | 1 (0,1) | 1 (0,1) |
| WWF | Ngo | 6 (4,2) | 2 (1,1) | 0 | 0 | 0 | 5 (3,2) | 0 | 1 (1,0) | 0 | 3 (2,1) |
| MWF | Gov | 5 (3,2) | 2 (1,1) | 0 | 2 (1,1) | 0 | 0 | 1 (1,0) | 0 | 0 | 0 |
| AZS | Acad | 4 (2,2) | 0 | 2 (1,1) | 2 (1,1) | 0 | 0 | 1 (1,0) | 0 | 1 (0,1) | 0 |
| UB | Acad | 3 (1,2) | 1 (1,0) | 1 (1,0) | 0 | 0 | 0 | 1 (0,1) | 0 | 1 (0,1) | 0 |
| MN | Ngo | 2 (1,1) | 0 | 0 | 0 | 1 (1,0) | 0 | 0 | 0 | 0 | 1 (0,1) |
| OC | Ngo | 2 (1,1) | 0 | 0 | 0 | 1 (1,0) | 0 | 0 | 1 (0,1) | 0 | 0 |
| CMSN | Acad | 1 (1,0) | 0 | 0 | 0 | 0 | 0 | 1 (1, 0) | 0 | 0 | 0 |

**S3 Table. Number of mentioning of interaction themes by individual stakeholders.** Values between brackets represent No. times the theme characterized the incoming and the outgoing ties respectively.

† Institutions that could not be interviewed for which relationships were imputed
