# Supplementary material for: Social network analysis and the implications for Pontocaspian biodiversity conservation in Romania and Ukraine: A comparative study
Source: PLoS One. 2020 Oct 23;15(10):e0221833. doi: 10.1371/journal.pone.0221833 (PMC7584225; doi:10.1371/journal.pone.0221833)
Supplement: S1 Text — (DOCX) [file pone.0221833.s006.docx]

**S1 Text. Social network analysis methods**

Two institutions could not be interviewed resulting in missing network data. We imputed the missing data using the imputation-by-reconstruction method [1]. The preconditions for employing this method are: 1) respondents shall be similar to non-respondents, and 2) the obtained description of the relational link (from the respondent) shall be reliable. A Chi-squared test revealed no significant differences in the distribution of weights of received relationships between the respondents and non-respondents (p-value = 0.98), meaning that respondents are similar to non-respondents. Furthermore, the confirmation rate (proportion of relational links described similarly by both nodes involved) was 84 % indicating that the descriptions of relational links (provided by the respondents) can be considered as reliable. Therefore, we used the reconstruction method to impute the missing ties in the network.

We calculated the basic network characteristics such as number of actors and relational ties, graph density and centralization using CRAN R package ‘igraph’ [2]. The mean shortest distance was calculated using the CRAN R package ‘tnet’ [3] because the ‘igraph’ package does not take edge weights into account when measuring the shortest distance. Graph density is the extent to which nodes are connected to each other in the network. It is calculated by dividing the number of existing ties by all the possible ties in a network [4, 5]. Network centralization is the extent to which certain actors are more connected in the network than the others [5, 6]. A centralized network is one where only one or few actors are having the majority of the ties. Such a network has a high overall centralization score (on a 0 to 1 scale, 0 being completely decentralized and 1 fully centralized). Shortest distance is a minimum number of steps that the nodes are away from each other in a network; in weighted networks the tie weights are taken under consideration [7]. We used frequency of contact as a measure of strength of the relationship and defined strong relationships as the weights higher or equal to 3 on a scale ranging from no contact to very frequent contact (S1 Text).

We measured the centrality of individual nodes using degree centrality and betweenness centrality values. Degree centrality is the number of connections a particular actor has with all the other actors in a network [8]. We calculated the degree of a node through an in-degree and out-degree values. In-degree of a node is the number of in-coming links to it from the other nodes in a network and the out-degree of a node is the number of out-going links from this node to the other nodes in a network [9]. Furthermore, we measured and used the node strength values (extension of the degree centrality to the sum of tie weights when analyzing weighted networks) to determine the size of the nodes in a sociogram [10-12]. Betweenness centrality measures the extent to which a node is among other nodes in a network [8]. For weighted networks the betweenness centrality measure is based on algorithm of shortest path distance [13, 14] which was lately further developed to integrate the cost of intermediary nodes in the formulae [7]. We calculated node-level statistics using the CRAN R package ‘tnet’ [3] which considers tie weights and corrects for the number of intermediary nodes. We regarded the central stakeholders as the ones with centrality scores higher than the third quartile threshold values [15-17].

We measured brokerage combining quantitative and qualitative approaches. Brokers are the nodes which are between other nodes in a network and have the power to control the flow of information [18-20]. Quantitatively, brokerage was measured through the betweenness centrality and the Burt’s constraint metric [18, 19]. Betweenness centrality locates the brokers structurally, with respect to all the other actors in the network. Burt’s constraint, however, is a local measure of brokerage based on the triadic closure principle. A node connecting two disconnected nodes in an incomplete triad has a power to broker. Such nodes have low Burt’s constraint score, i.e. their behavior is not constrained by the other disconnected nodes in a triad [20, 21]. Qualitatively, we examined the network narratives and searched for the evidence that the stakeholders are actually engaging in brokering behavior. Brokering behavior in the context of biodiversity conservation implies the mobilization of information, deliberation between different types of stakeholders and potentially the mediation through working groups to address conservation issues [22]. In our study, we regarded the stakeholders with high betweenness scores, which also accounted for low Burt’s constraint values, and were involved in brokering behavior as brokers. We used only the strong ties (≥ 3) to calculate betweenness centrality and Burt’s constraint metric as they reflect regular contacts. We calculated Burt’s constraint utilizing CRAN R package ‘igraph’ [2].

Finally, we used a null-model test to identify the presence of ‘network homophily’ in the network. ‘Network homophily’ is the selective linking between actors based on specific attributes, in our case the category of stakeholder institutes [23]. With a null-model test, we tested whether densities within and between stakeholder groups (defined by the stakeholder category) were significantly higher or lower than the random expectation. We randomly assigned nodes to the stakeholders proportional to the true network and subsequently assessed the stakeholder's within and between group densities replicated 1000 times, resulting in 1000 stakeholder group density values. We ranked the obtained 1000 random values from low to high and compared the actual within and between group densities to the randomized results. If the actual density values were larger than the upper or smaller than the lower 2.5% threshold value of the random distribution, we regarded the true within or between group densities to be significantly higher or lower than expected by random chance.

References

1. Stork D, Richards WD. Nonrespondents in communication network studies: Problems and possibilities. Group & Organization Management. 1992;17(2):193-209.

2. Csardi G, Nepusz T. The igraph software package for complex network research. InterJournal, Complex Systems. 2006;1695(5):1-9.

3. Opsahl T. Structure and evolution of weighted networks: Queen Mary, University of London; 2009.

4. Scott J. Social network analysis: A handbook. 1991.

5. Wasserman S, Faust K. Social network analysis: Methods and applications: Cambridge university press; 1994.

6. Freeman LC, Roeder D, Mulholland RR. Centrality in social networks: II. Experimental results. Social networks. 1979;2(2):119-41.

7. Opsahl T, Agneessens F, Skvoretz J. Node centrality in weighted networks: Generalizing degree and shortest paths. Social networks. 2010;32(3):245-51.

8. Freeman LC. Centrality in social networks conceptual clarification. Social networks. 1978;1(3):215-39.

9. Kleinberg JM, editor Authoritative sources in a hyperlinked environment. In Proceedings of the ACM-SIAM Symposium on Discrete Algorithms; 1998: Citeseer.

10. Barrat A, Barthélemy M, Vespignani A. Weighted evolving networks: coupling topology and weight dynamics. Physical review letters. 2004;92(22):228701.

11. Newman ME, Girvan M. Finding and evaluating community structure in networks. Physical review E. 2004;69(2):026113.

12. Opsahl T, Colizza V, Panzarasa P, Ramasco JJ. Prominence and control: the weighted rich-club effect. Physical review letters. 2008;101(16):168702.

13. Brandes U. A faster algorithm for betweenness centrality. Journal of mathematical sociology. 2001;25(2):163-77.

14. Dijkstra EW. A note on two problems in connexion with graphs. Numerische mathematik. 1959;1(1):269-71.

15. Grilli G, Garegnani G, Poljanec A, Ficko A, Vettorato D, De Meo I, et al. Stakeholder analysis in the biomass energy development based on the experts’ opinions: the example of Triglav National Park in Slovenia. Folia Forestalia Polonica. 2015;57(3):173-86.

16. Paletto A, Hamunen K, De Meo I. Social network analysis to support stakeholder analysis in participatory forest planning. Society & natural resources. 2015;28(10):1108-25.

17. Yamaki K. Applying social network analysis to stakeholder analysis in Japan’s natural resource governance: two endangered species conservation activity cases. Journal of Forest Research. 2017;22(2):83-90.

18. Burt RS. Structural holes and good ideas. American journal of sociology. 2004;110(2):349-99.

19. Burt RS. The social capital of structural holes. The new economic sociology: Developments in an emerging field. 2002;148:190.

20. Burt RS. Structural Holes: The Social Structure of Competition. Harvard University Press, Cambridge, MA. 1992.

21. Francis RA, Goodman MK. Post-normal science and the art of nature conservation. Journal for Nature Conservation. 2010;18(2):89-105.

22. Fazey I, Evely AC, Reed MS, Stringer LC, Kruijsen J, White PC, et al. Knowledge exchange: a review and research agenda for environmental management. Environmental Conservation. 2013;40(1):19-36.

23. Newman ME. The structure and function of complex networks. SIAM review. 2003;45(2):167-256.
