# Supplementary material for: Social network analysis and the implications for Pontocaspian biodiversity conservation in Romania and Ukraine: A comparative study
Source: PLoS One. 2020 Oct 23;15(10):e0221833. doi: 10.1371/journal.pone.0221833 (PMC7584225; doi:10.1371/journal.pone.0221833)
Supplement: S1 Appendix — (PDF) [file pone.0221833.s007.pdf]

S1 Appendix. Survey questions

Background

1. Organization name
2. Name of the person interviewed
3. Position of the person interviewed
4. Location
5. Date

Relationships for social network analysis (SNA)

6. Do you have Professional acquaintance/links with (name of stakeholder)?
7. What matters (topics) do you discuss with this stakeholder concerning Pontocaspian biodiversity management/conservation?

   7a. Do you exchange scientific data/information/knowledge/opinions/advices regarding the Pontocaspian biodiversity?
8. From the table below, how strong would you classify your professional acquaintance/links with this stakeholder?

Weight	Strength	Definition
**0**	Absent	We are never in contact with each other
**1**	Very weak	We have been in contact at some point in the past and foresee contact in the future
**2**	Weak	We are in contact incidentally, e.g. in case they have a joint project or they otherwise need each other. However, the rate of interaction is low and irregular
**3**	Strong	We are in contact regularly, on a monthly or quarterly basis
**4**	Very Strong	We are in contact very often, on a daily or weekly basis

9. Do you think you have enough contact or is it not sufficient?

   9a. If the contact is not sufficient what is the reason you are not in contact more often?

10. What are the areas/issues/partners that you would like to improve on (regarding this stakeholder)?

Knowledge transfer

11. What way(s) of gathering information do you use (most)?
12. What would make you change your approach?
13. Is there any information that you would like to have but have no access to?
14. Is there any information that you choose not to use on purpose?

The Pontocaspian biodiversity related information/data

15. Are you satisfied with the information you find generally?
16. What is lacking from the data/information to be of use to you?
17. Are you associated with any international or national projects on Pontocaspian biodiversity conservation?
